# Merkel Cell Carcinoma: Evolving Therapeutics, Continued Challenges

**DOI:** 10.1002/hed.70054

**Published:** 2025-09-27

**Authors:** Hunter A. Holley, Maria Lyons, Barry O'Sullivan, Neville Shine, Robbie Woods, Orla McArdle, James Paul O'Neill

**Affiliations:** ^1^ Department of Graduate Entry Medicine Royal College of Surgeons in Ireland (RCSI) Dublin Ireland; ^2^ Department of Otorhinolaryngology, Head and Neck Surgery Beaumont Hospital Dublin Ireland; ^3^ Department of Otolaryngology Head and Neck Surgery RCSI Dublin Ireland; ^4^ Department of Plastic Surgery Beaumont Hospital Dublin Ireland; ^5^ St. Luke's Radiation Oncology Network Dublin Ireland; ^6^ Beaumont RCSI Cancer Centre Beaumont Hospital Dublin Ireland

**Keywords:** immunotherapy, Merkel Cell Carcinoma, pathogenesis, prognosis, therapeutics

## Abstract

**Background:**

Merkel Cell Carcinoma (MCC) is a rare, aggressive neuroendocrine malignancy with rising incidence, influenced by ultraviolet (UV) radiation and Merkel cell polyomavirus (MCPyV).

**Methods:**

This review summarizes recent advances in MCC management, based on an analysis of current literature, focusing on immune checkpoint inhibitors (ICIs), viral status implications, and evolving multimodal treatment strategies.

**Results:**

MCPyV‐positive MCC has a median overall survival (OS) of 6.6 years compared to 1.2 years for virus‐negative cases. The 5‐year OS rate for localized MCC is approximately 50%. Historically, the 5‐year OS for metastatic MCC was ~14%, but has significantly improved with ICIs. First‐line treatment with avelumab achieved a median OS of 20.3 months and a 5‐year OS of approximately 26%. Pembrolizumab demonstrated a median OS of 24.3 months, a median progression‐free survival (PFS) of 9.3 months, and a durable response with a median duration of response (DOR) of 39.8 months.

**Conclusion:**

Despite advances, MCC recurrence rates remain high (16.4% local, 32.1% regional, 9.5% distant), necessitating vigilant long‐term surveillance. Future research should focus on optimizing combination therapies, identifying predictive biomarkers, and refining treatment sequencing to further improve survival and quality of life.

## 1 Background

Merkel Cell Carcinoma (MCC) is a rare, aggressive neuroendocrine carcinoma of the skin associated primarily with ultraviolet (UV) radiation and Merkel cell polyomavirus (MCPyV) infection [1, 2, 3]. Merkel cells, located in the basal layer of the epidermis, are specialized neuroendocrine cells involved in light‐touch sensation, making them integral to the skin's sensory function. MCC predominantly affects older adults, with a notably higher incidence in fair‐skinned and immunocompromised populations [3, 4, 5]. The incidence of MCC varies globally, with approximately 0.7 cases per 100,000 individuals annually in the United States and up to 2.5 cases per 100,000 in Australia, attributed largely to UV exposure [5, 6]. Epidemiological studies reveal a rising global incidence, potentially due to increased awareness and diagnostic improvements [7, 8].

In virus‐positive MCC (VP‐MCC), clonal viral integration leads to oncogenic protein expression, while virus‐negative MCC (VN‐MCC) involves UV‐related DNA mutations and a higher mutational burden [1, 5, 9]. Specifically, UV radiation induces cyclobutene pyrimidine dimers (CPDs) and 6–4 photoproducts (6‐4PPs), which disrupt normal DNA structure and replication, leading to mutations that can drive tumorigenesis [10]. These UV‐induced lesions result in DNA strand breaks, incorrect base pairing, and genomic instability, characteristic of VN‐MCC. Approximately 80% of MCC cases in the United States are MCPyV‐positive, whereas VN‐MCC prevails in high UV regions like Australia [5, 6, 11]. Patients with chronic inflammatory conditions, including diabetes and hepatitis B, may face a higher risk of aggressive MCC, highlighting an immune component in MCC pathogenesis [4, 12]. Moreover, immunosuppression from conditions like rheumatoid arthritis or chronic hepatitis increases MCC vulnerability due to weakened immune surveillance [2, 13].

Clinically, MCC typically presents as a rapidly growing, firm, red‐to‐violaceous nodule on sun‐exposed skin, most commonly in the head and neck. Due to its clinical similarity to other benign skin lesions, MCC is frequently misdiagnosed, making biopsy and immunohistochemical staining essential for accurate identification [5, 12, 14]. Diagnostic markers include cytokeratin 20 (CK20), alongside neuroendocrine markers like synaptophysin and chromogranin [7, 12]. Imaging can be used to identify local spread or nodal metastases, emphasizing MCC's aggressive behavior and metastatic potential, particularly in immunocompromised patients [2, 4].

Survival outcomes for MCC depend on disease stage, viral status, and immune competency. The 5‐year OS for localized MCC is ~50%, dropping to 35% with nodal involvement and further declining to ~14% for metastatic disease [15]. However, with the introduction of ICIs, survival rates have significantly improved. VP‐MCC is associated with better prognosis than VN‐MCC, with a median OS of 6.6 years versus 1.2 years, respectively [16]. Despite advancements in treatment, MCC still carries high recurrence risks, with 16.4% locally, 32.1% regionally, and 9.5% for distant recurrence [17].

Therapeutic strategies for MCC have evolved significantly, with the advent of immunotherapy. PD‐1/PD‐L1 inhibitors such as avelumab and pembrolizumab have become first‐line options for advanced MCC, demonstrating superior durability compared to chemotherapy. Avelumab has increased median OS to ~20.3 months, surpassing historical chemotherapy outcomes (12–18 months) [18]. Real‐world response rates range from 29.1% to 72.1%, reinforcing ICIs as the standard of care [5, 6, 7, 19]. While traditional chemotherapy remains an option, its efficacy is limited by short‐lived responses and greater toxicity [2, 12, 20]. Recent studies suggest ICIs may also lower recurrence risk and improve long‐term survival in high‐risk MCC, particularly VP‐MCC cases [12, 13].

Given the ongoing challenges in managing MCC, especially among immunosuppressed patients who demonstrate diminished responses to ICIs, there is a critical need to consolidate recent advancements and emerging therapeutic strategies [5, 14, 20]. This literature review synthesizes current research focused on biomarkers predictive of treatment response and novel preclinical models aimed at elucidating MCC biology. Additionally, we discuss optimization of immunotherapy regimens, explore promising combination therapies, and evaluate emerging predictive tools designed for improved patient stratification [9, 11].

## 2 Methods

### 2.1 Inclusion and Exclusion Criteria

Included studies focused on MCC's clinical and molecular characteristics, therapeutic interventions, prognostic factors, and survival outcomes. Inclusion criteria encompassed peer‐reviewed clinical trials, reviews, case studies, and observational studies with comprehensive data on MCC management. Exclusion criteria involved non‐human studies and publications outside the 5‐year window (2019–2024), with exceptions made for seminal studies relevant to MCC pathogenesis and treatment.

### 2.2 Search Strategy

**FIGURE 1:**
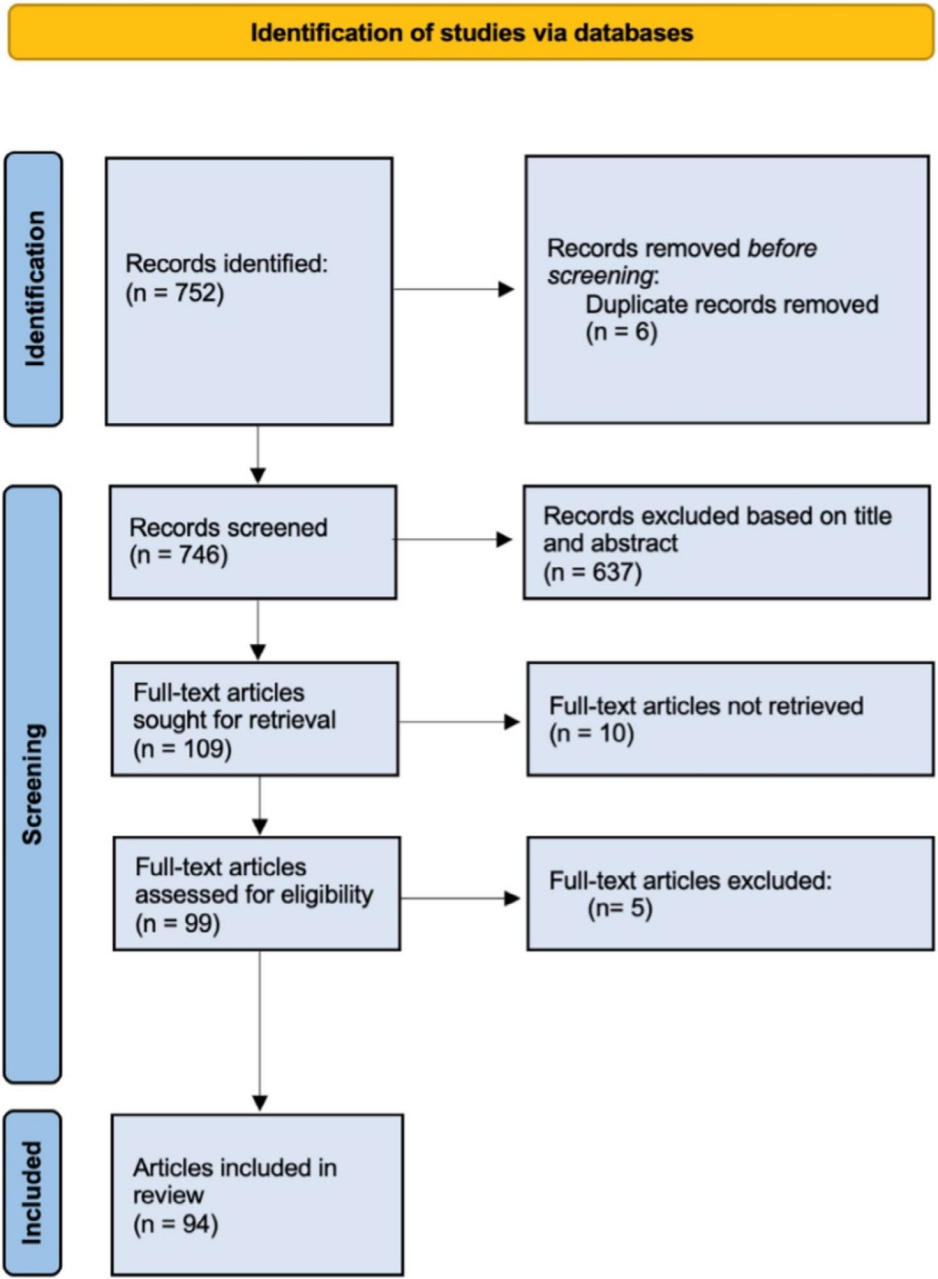
Adapted PRISMA flow diagram illustrating the literature search strategy. [Color figure can be viewed at wileyonlinelibrary.com]

This literature search was adapted from the PRISMA 2020 guidelines for clinical reviews [21]. Figure 1 demonstrates the search strategy and article selection process.

A comprehensive search was conducted across PubMed and Embase databases from October 28 to November 3, 2024. Keyword search terms included “Merkel Cell Carcinoma,” “immunotherapy,” “radiotherapy,” “molecular biology,” “surgery,” “epidemiology,” “risk factors,” “survival,” and “prognosis.” Boolean operators (AND, OR) were applied to optimize search specificity. Filters applied included publication within the last 5 years, full‐text availability, and study types restricted to peer‐reviewed clinical trials, reviews, case reports, randomized controlled trials, and meta‐analyses. Articles in English, German, and Spanish were included.

### 2.3 Study Selection and Data Extraction

Titles and abstracts were screened for relevance, followed by full‐text reviews of selected articles. Final articles underwent a systematic data extraction process to collect information on study design, population characteristics, key findings, and specific insights into MCC's clinical progression and treatment outcomes. Extracted data were synthesized into brief summaries to inform the evolving therapeutics and ongoing challenges in MCC management.

## 3 Discussion

### 3.1 Pathogenesis and Molecular Biology

MCC arises from a combination of viral, environmental, and immune‐related factors, with an estimated annual incidence of 0.7 per 100 000 people in the United States. Rates are significantly increased in regions with intense UV exposure, such as Australia, reflecting the role of environmental carcinogenesis in MCC development [10, 22, 23]. Advances in in vitro studies using organotypic epithelial raft cultures have replicated MCC's structural behavior, providing valuable insights into tumor biology and pathogenesis under controlled conditions [24].

MCPyV is integrated into the genome of approximately 80% of MCC cases, where viral oncoproteins, particularly the large T antigen, interfere with tumor suppressor proteins like p53 and RB1, driving tumorigenesis [25, 26]. Despite MCPyV being widespread in the general population—up to 80% of adults possessing antibodies—MCC predominantly occurs in older adults or immunocompromised individuals, reflecting the critical role of immune surveillance failure in tumor progression [26, 27, 28].

In contrast, MCPyV‐negative MCC cases—more common in regions with high UV exposure—exhibit a significantly higher mutational burden. These tumors are characterized by TP53 and RB1 mutations, which contribute to uncontrolled cell cycle progression, genomic instability, and higher metastatic potential [23, 29, 30]. UV exposure is a major driver of these mutations and also contributes to local immunosuppression, which may facilitate both viral replication and tumor initiation [23, 28, 29].

MCC disproportionately affects older Caucasian adults, with a median age of onset around 70 years, correlating with cumulative UV exposure and declining immune function [27, 28]. Certain immunosuppressed populations, including solid organ transplant recipients and individuals with HIV, face up to a 13‐fold increased risk of developing MCC [27, 28]. Similarly, chronic inflammatory conditions such as diabetes and hepatitis B may further compromise immune function, increasing susceptibility to MCC [18, 28]. Interestingly, MCC can also arise in areas of prior trauma or scarring, suggesting that localized immune alterations may influence tumor development [31].

### 3.2 Molecular and Epigenetic Regulation in MCC

MCC's distinction from other neuroendocrine malignancies is supported by specific molecular markers, including ATOH1, TFAP2B, and CEACAM6, which differentiate it from small‐cell lung carcinoma [32]. Histopathological analysis also reveals that MCC can coexist with other cutaneous malignancies, such as squamous cell carcinoma and basal cell carcinoma, particularly in immunocompromised patients. These “collision tumors” demonstrate abrupt histological transitions, suggesting shared pathogenic mechanisms with other skin cancers [33].

Choi et al. have recently identified BET (bromodomain and extra‐terminal domain) proteins, such as BRD4, as critical regulators of MCC cell proliferation. BET proteins bind to acetylated histones and promote transcription of oncogenic drivers, including MCPyV large T antigen and cell cycle regulators like Rb and E2F1 [34]. Targeting these pathways has emerged as a promising therapeutic approach, with BET degraders (e.g., BETd‐246) showing efficacy in disrupting these oncogenic mechanisms, particularly in treatment‐resistant MCC cases [34].

### 3.3 Surveillance

Circulating tumor DNA (ctDNA) analysis is a valuable non‐invasive biomarker for tracking tumor progression and recurrence, particularly in advanced MCC cases [35]. Given MCC's high recurrence potential—even decades after initial treatment—long‐term surveillance strategies must be tailored to patient demographics and tumor characteristics [36]. Currently, commercially available ctDNA assays provide individualized tests targeted toward tumor‐specific DNA alterations, enhancing sensitivity for recurrence detection and monitoring therapeutic responses. This represents an evolving, promising approach to clinical management [35, 36].

Reflecting MCC's unique molecular and histological profile, the 2022 World Health Organization (WHO) classification update recognizes MCC as a distinct neuroendocrine neoplasm, acknowledging both its overlap with other neuroendocrine tumors and its distinct molecular features [37, 38].

### 3.4 Presentation and Diagnosis

MCC often presents as a rapidly growing, painless nodule on sun‐exposed areas, such as the head and neck, which account for over 50% of cases [39, 40, 41]. Its visual resemblance to benign lesions like cherry angiomas and malignancies, such as basal and squamous cell carcinoma complicates timely diagnosis. Koumaki et al. noted these similarities, further highlighting the need for comprehensive clinical evaluation and histological confirmation [42].

Advanced diagnostic tools, such as dermoscopy and reflectance confocal microscopy (RCM), now aid in distinguishing MCC by identifying features like a milky red background, polymorphous vascular patterns, and hypo‐reflective cell clusters [40]. However, imaging challenges persist, particularly in cases where poly‐L‐lactic acid fillers can complicate fluorodeoxyglucose positron emission tomography scan (FDG‐PET) interpretations, leading to potential diagnostic confusion [43]. MCC may also coexist with other malignancies, such as squamous cell carcinoma, resulting in combined presentations with abrupt histological transitions, highlighting the importance of thorough histopathological analysis as the gold standard for diagnosis [39].

Histologically, MCC tumors typically exhibit small, basophilic neuroendocrine cells and a high nuclear‐to‐cytoplasmic ratio, forming sheets or nests in the dermis [39]. Immunohistochemistry (IHC) plays a vital role in confirming MCC, particularly in complex cases where it may coexist with hematologic malignancies (i.e., acute myeloid leukemia) or other skin cancers [39]. MCC tumors typically express CK20 in a dot‐like perinuclear staining pattern and neuroendocrine markers like synaptophysin and chromogranin [39, 44]. In complex cases, CK20 and CD56 markers are vital for accurately distinguishing MCC from other neoplasms [44, 45]. The presence of large T antigen associated with MCPyV further supports MCC's viral‐driven pathogenesis [25].

### 3.5 Interventions, Survival, and Prognosis

The management of MCC necessitates a multimodal approach, integrating surgical resection, radiotherapy, immunotherapy, and systemic chemotherapy based on disease stage, tumor burden, and patient‐specific factors. Localized MCC is primarily treated with surgery, often supplemented by adjuvant radiotherapy to improve locoregional control, whereas metastatic or unresectable disease is best managed with immune checkpoint inhibitors, which have largely supplanted chemotherapy due to superior efficacy and durability of response. However, chemotherapy remains a consideration in select scenarios, such as rapid cytoreduction in symptomatic disease or in combination regimens for refractory cases. As treatment strategies evolve, optimizing therapeutic selection requires balancing oncologic control, toxicity profiles, and functional outcomes. The following sections provide a detailed analysis of each modality, with emphasis on comparative efficacy, indications, and emerging therapeutic synergies.

**FIGURE 2:**
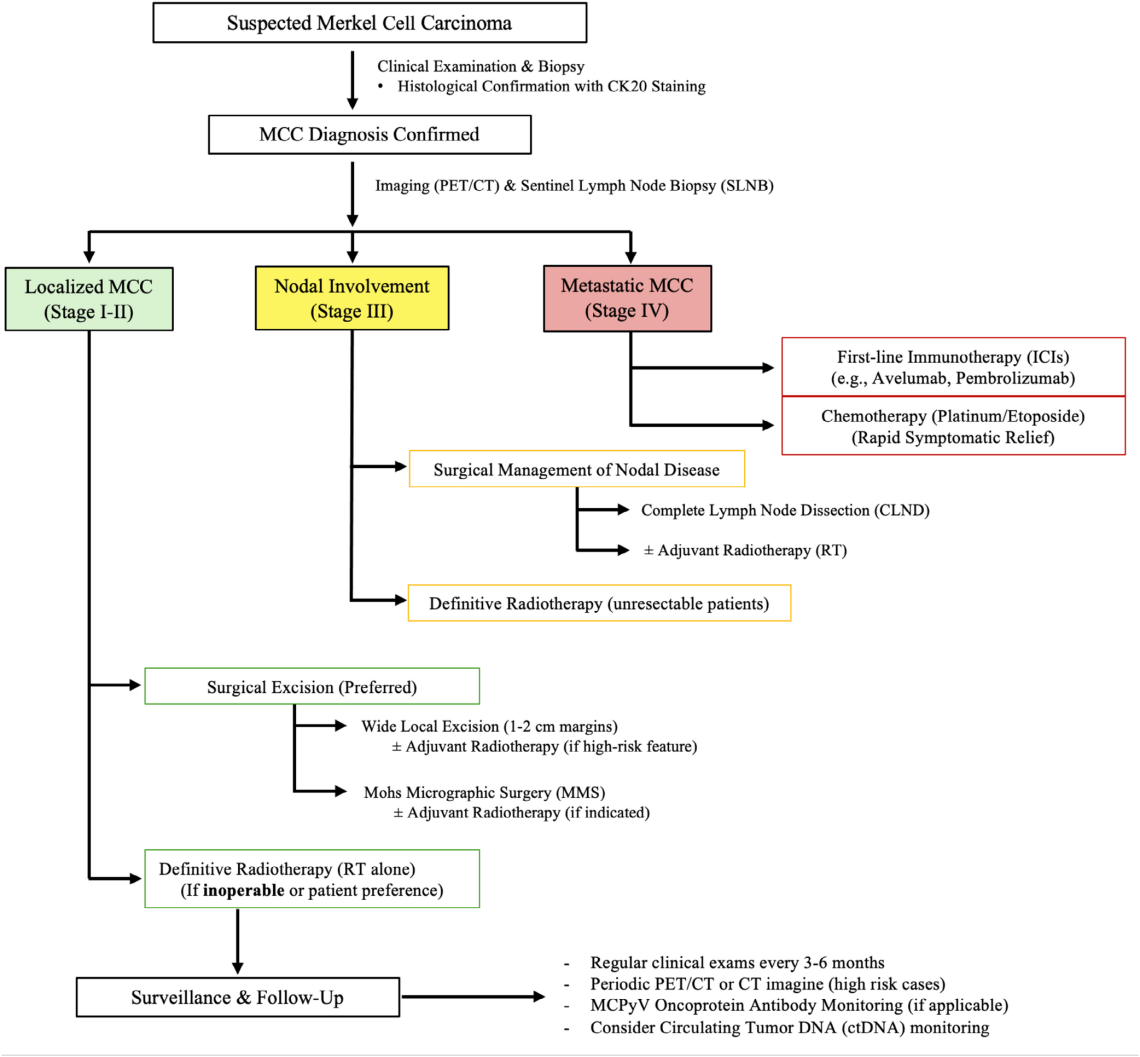
Management algorithm for Merkel Cell Carcinoma (MCC). [Color figure can be viewed at wileyonlinelibrary.com]

Figure 2 provides a structured management algorithm for MCC, illustrating evidence‐based therapeutic pathways tailored to disease stage, extent of nodal involvement, and metastatic status.

#### 3.5.1 Surgery

Surgical intervention remains a cornerstone in the management of primary MCC, with approaches designed to optimize local control while preserving cosmetic and functional outcomes. Historically, surgical intervention for localized MCC has demonstrated a 5‐year overall survival (OS) rate of approximately 50%, decreasing significantly to around 35% when regional nodes are involved, and further decreasing to 14% with distant metastases [46, 47]. Wide Local Excision (WLE) and Mohs Micrographic Surgery (MMS) represent the two primary strategies, each offering distinct benefits based on patient‐specific and tumor‐specific factors.

WLE is recommended with margins of 1–2 cm to ensure adequate clearance of malignant cells, particularly in high‐risk areas [48]. However, surgery alone may be insufficient for cases with perineural invasion, lymphovascular involvement, or positive margins. As surgery does not address residual microscopic disease, a multimodal approach incorporating adjuvant radiotherapy is often necessary to improve local control and reduce recurrence in 80%–90% of cases within the first 2 years following initial treatment [46]. Recurrence typically occurs early, with a median time between 7 and 9 months post‐treatment [46].

In cosmetically sensitive regions such as the head and neck, MMS is often preferred for its precision in margin control and tissue preservation. A multicenter cohort study by Sharma and Demer compared WLE and MMS outcomes and found no significant difference in overall survival, suggesting that either technique may be appropriate depending on the anatomical site and individual case considerations [49]. Paraffin‐embedded Margin‐controlled MMS (PMMS), an advanced variation of MMS, has demonstrated superior precision in assessing tumor margins, which is critical for reducing recurrence rates [45, 50]. This enhanced accuracy makes PMMS particularly advantageous for MCC patients where margin control is paramount to achieving optimal oncologic and cosmetic outcomes.

For localized MCC, achieving optimal disease control requires a surgical approach, with adjuvant radiotherapy considered in cases of high‐risk features or incomplete resection. When surgical resection is not feasible or a patient is deemed a poor surgical candidate, radiotherapy alone may be an alternative approach [51]. Studies indicate that combining surgical excision with radiotherapy may lead to better outcomes, particularly in cases with a high risk of locoregional spread.

Sentinel lymph node biopsy (SLNB) is routinely recommended at the time of surgical excision due to MCC's aggressive nature and high rate of occult nodal metastasis, even among small primary tumors. Approximately 24%–32% of clinically node‐negative MCC patients harbor occult nodal disease detectable by SLNB, and even small MCC lesions (≤ 1 cm) exhibit a significant SLNB positivity rate (14%) [52]. Thus, SLNB remains a critical staging tool, distinguishing MCC from other cutaneous malignancies with comparatively lower occult nodal involvement [53]. By accurately assessing regional lymph node involvement, SLNB effectively guides therapeutic decisions such as the addition of radiotherapy or systemic therapies [52, 53].

#### 3.5.2 Radiotherapy

Radiotherapy (RT) is integral to MCC treatment, used both as adjuvant therapy after surgery and as a primary option when resection is not feasible. Following excision, RT improves local control and reduces recurrence risk, particularly in cases with lymphovascular invasion or positive margins. As a standalone treatment, RT provides durable disease control but lacks the immediate tumor removal achieved with surgery, reinforcing the need for individualized treatment planning. Adjuvant RT is especially valuable in patients with high‐risk features, where it enhances survival and significantly lowers the likelihood of local recurrence [54].

For advanced disease or oligometastatic progression, stereotactic body radiation therapy (SBRT) has emerged as a precise and effective option. SBRT allows targeted delivery of high‐dose radiation to metastatic lesions while sparing surrounding healthy tissues, making it particularly beneficial in patients resistant to immunotherapy or those with limited metastatic disease [55].

The role of RT is further emphasized in MCC involving the head and neck, where recurrence rates are notably higher without intervention and patterns of recurrence are typically locoregional. The ability of radiation to eradicate occult microscopic disease is of particular value in the head and neck region, as its use may spare the patient a more extensive surgical procedure associated with significant morbidity. Irradiation of the neck may allow omission of dissection for clinically uninvolved necks, reducing overall treatment toxicities. Data support the use of RT in early‐stage, low‐risk cases of pathologic stage I MCC to reduce recurrence risk and achieve local control [56]. However, this site‐specific approach highlights the need to balance therapeutic benefit with the risk of unnecessary radiation exposure, particularly in lower‐risk regions.

Emerging studies suggest that RT may complement immunotherapy by modifying the tumor microenvironment, potentially enhancing tumor antigen presentation and improving treatment response [57]. This synergy has led to increasing interest in combining RT with systemic therapies, particularly immune checkpoint inhibitors like avelumab, which has demonstrated efficacy in MCC. Preclinical and clinical data indicate that RT may enhance avelumab's efficacy by priming the immune system, increasing tumor infiltration by activated T cells, and potentially improving long‐term disease control [58, 59]. As research into these interactions evolves, ongoing clinical trials aim to define optimal treatment strategies that integrate RT with systemic therapies, further refining its role in MCC management [60, 61].

#### 3.5.3 Chemotherapy and Emerging Therapeutic Strategies

While chemotherapy has historically been a mainstay in treating metastatic MCC, its role has diminished in favor of immunotherapy and targeted treatments. Conventional chemotherapeutic agents, such as platinum‐based drugs and topoisomerase inhibitors like etoposide, have demonstrated initial response rates but yield less durable responses compared to immunotherapy and are associated with greater systemic toxicity [62]. Historically, patients with metastatic MCC treated with chemotherapy alone experienced limited outcomes, with median overall survival typically ranging from 12 to 18 months [46]. However, in rapidly progressive disease, where immediate reduction of tumor burden is critical and time sensitive, chemotherapy remains a valuable short‐term intervention.

For patients with locally advanced MCC, where systemic chemotherapy may not be appropriate, isolated limb infusion (ILI) has emerged as an alternative strategy [63]. This regional technique delivers high‐dose cytotoxic agents directly to affected extremities, minimizing systemic toxicity while effectively treating in‐transit disease. ILI has been particularly beneficial for patients with unresectable limb lesions, providing tumor control without significant systemic side effects.

In parallel, the detection of MCPyV in MCC has also spurred the development of virus‐specific therapies. Novel techniques such as siRNA‐based gene silencing and CRISPR/Cas9 editing have been proposed to target MCPyV oncogenes, offering a potential precision medicine approach [64]. Although still experimental, these strategies represent an exciting avenue for developing targeted antiviral therapies for VP‐MCC.

Beyond viral‐directed treatments, the immune microenvironment of MCC tumors has become a focus of research. Studies have identified tertiary lymphoid structures within MCC tumors, suggesting distinct immune profiles between virus‐positive and virus‐negative cases [65]. These findings provide a rationale for developing personalized immunotherapies tailored to a patient's tumor immune composition, moving beyond a one‐size‐fits‐all approach.

Nodal MCC without a known primary site presents another clinical scenario where chemotherapy remains relevant. Recent findings by Fazio et al. indicate that patients with isolated regional nodal involvement may benefit from chemotherapy, particularly when integrated with radiotherapy and immunotherapy to achieve long‐term disease control [66].

Neoadjuvant approaches have also emerged as a promising avenue in MCC management. Preoperative administration of systemic treatments, such as ICIs, has shown potential in reducing tumor burden before definitive surgery [67, 68]. This approach not only enhances resectability in high‐risk patients but also delivers prolonged immune‐mediated responses, which are not typically seen with chemotherapy alone.

In summary, while chemotherapy remains an option for MCC patients unable to tolerate immunotherapy, its role is increasingly adjunctive rather than primary. Future treatment strategies will likely focus on multimodal approaches, integrating immunotherapy, targeted therapies, and novel viral‐directed treatments, reflecting a deeper understanding of MCC's molecular and immune landscape [69, 70].

#### 3.5.4 Immunotherapy

Immunotherapy has revolutionized MCC management by offering more durable responses and fewer adverse effects compared to chemotherapy. The pivotal JAVELIN Merkel 200 study reported that first‐line treatment with avelumab achieved a median OS of 20.3 months and a notable 5‐year OS rate of approximately 26%, considerably surpassing traditional chemotherapy results [18]. Similarly, the KEYNOTE‐913 trial evaluating pembrolizumab as first‐line therapy reported an objective response rate (ORR) of 49%, a median OS of 24.3 months, and a promising 24‐month OS rate of 51%, with durable responses lasting a median of 39.8 months and approximately 69% of responders maintaining their response at 24 months [71]. These outcomes reinforce the substantial survival benefit associated with early initiation of ICIs in MCC management.

Avelumab has emerged at the forefront of this transformation. The JAVELIN Merkel 200 study demonstrated an ORR of approximately 39.7%, with sustained clinical responses and manageable safety observed at a 4‐year follow‐up [72, 73]. Real‐world studies have reinforced these findings, demonstrating a 57% ORR with complete responses observed in 29% of patients treated with avelumab, and particularly high efficacy (83%) in patients with limited metastatic sites [74]. Additionally, factors such as patient age and body mass index have shown no significant correlation with response rates or survival outcomes, highlighting the broad applicability of immunotherapy across diverse patient populations [75].

MCPyV cases typically exhibit better survival outcomes than virus‐negative cases, likely due to increased immunogenicity, rendering these tumors more responsive to immunotherapy. This difference underscores the importance of considering viral status when selecting treatment approaches [12, 13, 16].

Beyond their use in metastatic disease, ICIs have shown promise in neoadjuvant settings. In the CheckMate 358 trial, neoadjuvant nivolumab reduced tumor burden, achieving pathological downstaging in approximately 47.2% of patients with resectable MCC, thus facilitating subsequent surgical intervention [76]. For patients unresponsive to initial ICIs such as avelumab, alternative ICIs, such as pembrolizumab, provide viable options. Both agents target the PD‐1/PD‐L1 pathway; however, their mechanisms differ—avelumab blocks PD‐L1 on tumor cells, indirectly reactivating T‐cells, while pembrolizumab directly inhibits PD‐1 receptors on T‐cells, preventing their inactivation [77]. This distinction may explain the efficacy observed in pembrolizumab‐treated patients within the KEYNOTE‐913 study, which reported favorable response rates in recurrent and locally advanced MCC [71]. In certain cases, combining ICIs with radiotherapy or chemotherapy has demonstrated synergistic effects, leading to tumor regression and improved treatment efficacy [77].

Adjuvant immunotherapy is also gaining momentum for high‐risk MCC patients. The ADMEC‐O trial demonstrated improved disease‐free survival (DFS) with the PD‐1 inhibitor, nivolumab, compared to observation following complete surgical resection, with 12‐ and 24‐month DFS rates of 85% and 84%, respectively, versus 77% and 73% in the observation group [78]. This corresponded to absolute risk reductions of 9% and 10% at 1 and 2 years [78]. These findings underscore immunotherapy's potential to prevent recurrence and optimize outcomes in high‐risk, localized MCC cases.

Immunotherapy's ability to target the immune response against MCPyV has been substantiated by studies identifying oncoprotein antibody titers as reliable markers for recurrence detection. Alexander et al. highlighted rising antibody levels as an early indicator of hidden MCC recurrences, facilitating timely intervention [79]. Additionally, Nishizawa et al. demonstrated immunotherapy's effectiveness in complex cases, such as MCC coexisting with other malignancies like follicular lymphoma, further underscoring its versatility in challenging presentations [80].

For treatment‐refractory MCC, ICIs such as nivolumab and ipilimumab have demonstrated efficacy, especially when combined with stereotactic body radiation therapy (SBRT), highlighting a potential synergy in managing advanced, MCPyV‐positive tumors [81, 82]. Alternative salvage therapies, including CTLA‐4 inhibitors like ipilimumab, have been explored in cases resistant to standard anti‐PD(L)1 therapy, though with variable clinical efficacy [83, 84].

The synergy between immunotherapy and other treatment modalities, such as radiotherapy and chemotherapy, is being explored to enhance treatment outcomes. Combined approaches, including avelumab with radiotherapy, have demonstrated tumor regression in advanced MCC, indicating the potential for multimodal therapies to overcome resistance and improve survival [77]. Emerging data suggest that SBRT may enhance immune responses in patients resistant to checkpoint inhibitors, highlighting the value of integrating immunotherapy with localized treatments.

Further research into the immune microenvironment of MCC tumors offers additional opportunities for therapeutic optimization. Factors such as T‐cell infiltration and tumor‐associated macrophage activity play crucial roles in immunotherapy responsiveness [85]. By better understanding these mechanisms, novel strategies can be developed to improve patient outcomes.

In specific anatomical locations, such as periorbital MCC, immunotherapy poses unique challenges due to the delicate tissue involved. Localized approaches, including precision drug delivery systems, are being investigated to target tumors effectively while sparing healthy structures [86, 87]. These advancements highlight the growing focus on personalized treatment strategies for MCC.

ICIs like avelumab and pembrolizumab have become central to MCC management, offering durable responses and significant survival benefits. Additionally, biomarker analyses indicate that PD‐L1 expression and the absence of prior chemotherapy correlate with improved responses to ICIs [18]. However, immunosuppressed patients with MCC exhibit reduced responses to immunotherapy, underscoring the need for alternative strategies in these populations [16]. Ongoing research continues to refine optimal treatment sequencing, combination strategies, and biomarker‐driven patient selection, paving the way for more personalized and effective treatments [88, 89].

### 3.6 Viral Status and Disease Markers

MCPyV status significantly influences prognosis. VP‐MCC patients generally have better survival outcomes than VN‐MCC patients (median survival 6.6 vs. 1.2 years, respectively) [47]. VP‐MCC tumors have fewer genetic mutations, as their oncogenesis is driven by viral oncoproteins, whereas VN‐MCC tumors exhibit high mutational burdens from UV‐induced DNA damage [16, 46, 47]. This distinction may impact treatment response, as VP‐MCC tumors often respond better to ICIs [47]. Table 1 summarizes key clinical, molecular, and therapeutic differences between VP‐MCC and VN‐MCC, highlighting essential considerations for prognosis and treatment planning.

**TABLE 1:** Comparative clinical and molecular features of viral‐positive (VP‐MCC) versus viral‐negative Merkel Cell Carcinoma (VN‐MCC) and their therapeutic implications.

Feature	VP‐MCC	VN‐MCC
Virus Association	Merkel cell polyomavirus (MCPyV)‐associated	UV‐induced, MCPyV‐negative
Mutation Burden	Lower mutation burden	Higher mutation burden
Common Mutation	Rare driver mutations; absence of TP53 and RB1 mutations	Frequent TP53 and RB1 mutations
Clinical Presentation	Often occurs in immunosuppressed populations (e.g., HIV, transplant recipients)	Strongly linked to sun exposure; occurs in older, fair‐skinned individuals
Immune Microenvironment	High infiltration of CD8+ T‐cells; high levels of immunosuppressive M2 macrophages; elevated PD‐L1 expression	Lower CD8+ T‐cell density; fewer M2 macrophages
Prognosis	More favorable prognosis; median OS ~6.6 years	Poorer prognosis; median OS ~1.2 years
Survival Rates (Stage‐specific)	Improved outcomes; 5‐year OS localized MCC ~50%; nodal MCC ~35%	Poorer outcomes overall; higher recurrence rates
Response to Immunotherapy (ICIs)	Durable responses; less influenced by TMB (ORR ~40%–50%; Median OS ~20–24 months)	Potentially higher response rates due to high TMB; response less predictable
Typical Treatment Strategies	Excellent and durable response to ICIs; superior outcomes compared to chemotherapy	ICIs effective, but chemotherapy and combined modalities sometimes needed; shorter duration of responses observed
Emerging Treatments	MCPyV‐directed therapies (e.g., CRISPR, siRNA) show potential	Focus on genomic profiling; combination therapies often required

Advanced imaging techniques, such as PET‐CT, and biomarker tracking (e.g., viral oncoprotein antibody levels), are emerging as tools for predicting survival and monitoring disease progression. Studies suggest that rising antibody titers may serve as early indicators of MCC recurrence, enabling proactive intervention [47]. Additionally, genomic profiling is refining treatment selection, facilitating personalized therapeutic approaches based on tumor molecular and immune characteristics [46, 90].

### 3.7 Impact of Treatment Modalities on MCC Survival

MCC treatment decisions are guided by tumor biology, disease extent, and patient‐specific factors, all of which influence survival outcomes. As discussed in Survival & Prognosis, early detection and multimodal treatment strategies significantly improve survival [18, 46]. Furthermore, as highlighted in Viral Status and Disease Markers, VP‐MCC and VN‐MCC exhibit distinct biological behaviors, which may impact treatment response. VP‐MCC tumors, driven by viral oncoproteins, often demonstrate enhanced sensitivity to ICIs, whereas VN‐MCC tumors, which exhibit a higher mutational burden, may exhibit variable responses [12, 13, 47].

For localized MCC, surgery remains the primary treatment, particularly when combined with SLNB to evaluate regional spread. Adjuvant RT has been shown to significantly improve recurrence‐free survival and OS in high‐risk cases, including patients with large primary tumors (> 1 cm), chronic immunosuppression, or positive SLNB findings [46]. A multimodal approach integrating surgery, SLNB, and RT has demonstrated superior local relapse‐free and distant metastasis‐free survival outcomes, reinforcing its role in early‐stage disease control [91].

For advanced or metastatic MCC, ICIs have replaced chemotherapy as the standard of care, offering superior survival benefits. As highlighted in Survival & Prognosis, first‐line avelumab therapy has increased median OS to ~20.3 months, a notable improvement over chemotherapy [18, 47]. Additionally, avelumab and pembrolizumab have demonstrated prolonged progression‐free survival (PFS) and OS, particularly in VP‐MCC patients, reinforcing the importance of immune‐based treatment approaches. Recent real‐world and clinical studies indicate that patients receiving ICIs achieve a median PFS of 16.8 months and OS of 20.6 months, underscoring their effectiveness in advanced MCC [15, 47].

Additionally, newer immunotherapies are expanding treatment options. Retifanlimab, a PD‐1 receptor‐blocking antibody, has been approved for metastatic or recurrent locally advanced MCC, further diversifying the immunotherapy landscape [92]. Ongoing trials continue to evaluate combination strategies (e.g., ICIs + RT or chemotherapy) to optimize treatment durability and reduce resistance. These advancements mark a fundamental shift in MCC management, underscoring the importance of integrating viral status, tumor biology, and multimodal treatment strategies to improve long‐term survival.

Tables 2 and 3 comprehensively summarize key therapeutic modalities in MCC, systematically integrating landmark clinical trial evidence, survival and efficacy data, and practical considerations for clinical decision‐making. Treatments are categorized clearly based on disease stage, highlighting local and regional interventions (Table 2) versus systemic therapies for advanced MCC (Table 3), thereby offering a structured guide to inform personalized therapeutic strategies.

**TABLE 2:** Local and regional treatment modalities in Merkel Cell Carcinoma (MCC).

Treatment modality	Indications	Efficacy/survival data	Citation	Advantages	Limitations
Surgery (Wide Local Excision + SLNB)	Localized MCC, small tumors without metastases	5‐year OS ~50% for localized MCC; recurrence rates: 16.4% local, 32.1% regional	Fu et al., J Invest Dermatol, 2022 (meta‐analysis, 926 cases) [17]	Immediate tumor removal reduces recurrence risk	High recurrence even with clear margins
Adjuvant Radiotherapy (RT)	Post‐surgical MCC, high‐risk cases (large tumors, positive margins, nodal involvement)	Improves recurrence‐free survival; lowers locoregional recurrence	Becker et al., Onkol. 2024 (retrospective study) [5]	Enhances local control, reduces recurrence risk	Does not prevent distant metastases, potential toxicity
Definitive RT (without surgery)	Unresectable MCC, elderly, or poor surgical candidates	Local relapse rate: 13.7%, comparable efficacy to surgery + RT in select cases	Dubois et al., Radiat Oncol. 2021 (retrospective trial) [88]	Non‐invasive, viable for non‐surgical patients	Lower long‐term control vs. surgery
Neoadjuvant ICIs (Nivolumab)	Neoadjuvant/adjuvant for high‐risk resectable MCC	Pathologic CR ~47%; DFS 85% (12 months), 84% (24 months) vs. 77%, 73%	Topalian et al., 2020 (CheckMate 358 trial) vs. (ADMEC‐O) [89]	Shrinks tumors pre‐surgery, improves outcomes	Not yet standard of care, further validation needed

**TABLE 3:** Systemic therapies for advanced Merkel Cell Carcinoma (MCC).

Treatment modality	Indications	Efficacy/Survival data	Citation	Advantages	Limitations
Immunotherapy (ICIs: Avelumab, Pembrolizumab)	First‐line for metastatic MCC	5‐year OS: 26%, Median OS: 12.6 months (Avelumab); ORR 56%, Median PFS: 16.8 months (Pembrolizumab)	D'Angelo et al., 2024 (JAVELIN Merkel 200); Lessans et al., 2024 (KEYNOTE‐017) [18, 89]	Durable responses, superior to chemotherapy, effective in VP‐MCC	Resistance in some patients, lower efficacy in immunosuppressed individuals
Chemotherapy (Platinum‐based + Etoposide)	Rapid tumor burden reduction (e.g., airway/spinal cord compression)	ORR 53%–76%, median duration of response ~3 months, median PFS ~3–8 months (pre‐immunotherapy era)	Lewis et al., Dermatol Clin. 2023 (review) [12]	Provides immediate tumor shrinkage	High toxicity, short‐lived responses, less effective than ICIs
Retifanlimab (PD‐1 Inhibitor)	Metastatic/recurrent locally advanced MCC	ORR: 52.3% (CR: 18.5%, PR: 33.8%), DCR: 61.5%; FDA‐approved March 2025	Kang, Drugs 2023 (POD1UM‐201 trial) [92]	Expands immunotherapy options	Limited long‐term data
Combination Strategies (ICIs + RT or Chemo)	High‐risk MCC, recurrent disease	Potential synergy improving PFS/OS	Tanda et al., Front Oncol. 2021 (systematic review) [88]	May enhance responses, overcomes resistance	Ongoing research is needed for optimal use
Stereotactic Body Radiation Therapy (SBRT)	Oligometastatic MCC, brain metastases	Provides control of metastatic lesions	Tanda et al., Front Oncol. 2021 (systematic review); Lessans et al., Curr Oncol Rep. 2024 (review) [88, 89]	Precision‐targeted therapy spares surrounding tissues	Some lesions are not suitable for SBRT

### 3.8 Neuro‐Metastatic MCC and Risk of Recurrence

Neuro‐metastatic MCC represents a particularly aggressive subset, with survival rates influenced by neurological involvement and immune status. Harary et al. reported an average survival of less than 12 months, although neurosurgical resection combined with radiotherapy and immunotherapy provided survival benefits in select cases, particularly for solitary brain metastases [93]. Combined radio‐immunotherapy approaches have shown promise, demonstrating complete clinical regression in advanced presentations [46].

Recurrence remains a significant concern across all stages of MCC. Even with clear surgical margins, recurrence rates remain high: A meta‐analysis involving 926 cases showed that the local recurrence rate (LRR) for patients with pathologically clear margins was 16.4%, while the in‐transit recurrence rate (ITR) was 9.5%, regional nodal recurrence rate (RRR) was 32.1%, and distant recurrence rate (DRR) was 9.5% [17]. These recurrence patterns may reflect the biological aggressiveness of MCC and its propensity for local extension [46]. Patients with MCC are also at elevated risk for secondary malignancies, with a meta‐analysis showing a standardized incidence ratio (SIR) of 1.52 overall and an SIR of 3.09 for malignant melanoma, emphasizing the need for ongoing surveillance post‐treatment [94].

Despite these advancements, challenges remain in optimizing MCC management. The heterogeneity of MCC, particularly the distinct molecular profiles of virus‐positive and virus‐negative cases, complicates treatment standardization. Additionally, while immune checkpoint inhibitors have transformed treatment paradigms, a subset of patients remains resistant to therapy, underscoring the need for biomarker‐driven, personalized approaches. Furthermore, the lack of large‐scale, randomized clinical trials specific to MCC limits the generalizability of current findings, necessitating further research into predictive markers of treatment response. Ongoing studies into the immune microenvironment, ctDNA monitoring, and novel therapeutic targets will be critical for advancing patient outcomes.

### 3.9 Emerging Research and Precision Oncology

Emerging research focuses on the immune microenvironment of MCC, including the role of T‐cell infiltration and tumor‐associated macrophages in shaping immunotherapy responses [90]. Tertiary lymphoid structures within tumors may correlate with improved immunotherapy outcomes, offering insights into patient‐specific prognostic factors and guiding precision treatment strategies [47]. Advances in genomic and molecular profiling continue to drive personalized approaches, enabling clinicians to predict responses to immunotherapy and radiotherapy with greater accuracy [46, 95].

In summary, survival outcomes for MCC have improved with advancements in multimodal therapies, particularly immunotherapy and molecular profiling. While localized MCC benefits from surgery and adjuvant RT, metastatic disease now relies on ICIs as standard of care. Comprehensive surveillance and further research into the molecular underpinnings of MCC will continue to refine treatment protocols, offering hope for improved prognosis and quality of life in this aggressive malignancy.

## 4 Conclusion

MCC is an aggressive neuroendocrine malignancy with rising global incidence, driven by MCPyV integration and UV‐induced mutagenesis. While surgery with SLNB and adjuvant RT remains critical for localized disease, immune ICIs have redefined the management of advanced MCC, particularly in virus‐positive cases. However, high recurrence rates and limited efficacy in virus‐negative and immunotherapy‐resistant disease highlight the need for novel strategies.

Molecular profiling, ctDNA monitoring, and immune‐based interventions are shaping precision medicine approaches that may improve therapeutic selection, predict resistance, and enhance long‐term outcomes. Future efforts must focus on refining combination therapies, identifying predictive biomarkers, and overcoming treatment resistance to further optimize MCC management and survival.

## Data Availability

Data sharing not applicable to this article as no datasets were generated or analysed during the current study.
